# Atypical miRNA expression in temporal cortex associated with dysregulation of immune, cell cycle, and other pathways in autism spectrum disorders

**DOI:** 10.1186/s13229-015-0029-9

**Published:** 2015-06-19

**Authors:** Bradley P. Ander, Nicole Barger, Boryana Stamova, Frank R. Sharp, Cynthia M. Schumann

**Affiliations:** Department of Psychiatry & Behavioral Sciences, MIND Institute, University of California at Davis Medical Center, 2805 50th Street, Sacramento, CA 95817 USA; Department of Neurology, MIND Institute, University of California at Davis Medical Center, 2805 50th Street, Sacramento, CA 95817 USA

**Keywords:** Autism, miRNA, snoRNA, Auditory cortex, Superior temporal sulcus, Temporal lobe

## Abstract

**Background:**

Autism spectrum disorders (ASDs) likely involve dysregulation of multiple genes related to brain function and development. Abnormalities in individual regulatory small non-coding RNA (sncRNA), including microRNA (miRNA), could have profound effects upon multiple functional pathways. We assessed whether a brain region associated with core social impairments in ASD, the superior temporal sulcus (STS), would evidence greater transcriptional dysregulation of sncRNA than adjacent, yet functionally distinct, primary auditory cortex (PAC).

**Methods:**

We measured sncRNA expression levels in 34 samples of postmortem brain from STS and PAC to find differentially expressed sncRNA in ASD compared with control cases. For differentially expressed miRNA, we further analyzed their predicted mRNA targets and carried out functional over-representation analysis of KEGG pathways to examine their functional significance and to compare our findings to reported alterations in ASD gene expression.

**Results:**

Two mature miRNAs (miR-4753-5p and miR-1) were differentially expressed in ASD relative to control in STS and four (miR-664-3p, miR-4709-3p, miR-4742-3p, and miR-297) in PAC. In both regions, miRNA were functionally related to various nervous system, cell cycle, and canonical signaling pathways, including PI3K-Akt signaling, previously implicated in ASD. Immune pathways were only disrupted in STS. snoRNA and pre-miRNA were also differentially expressed in ASD brain.

**Conclusions:**

Alterations in sncRNA may underlie dysregulation of molecular pathways implicated in autism. sncRNA transcriptional abnormalities in ASD were apparent in STS and in PAC, a brain region not directly associated with core behavioral impairments. Disruption of miRNA in immune pathways, frequently implicated in ASD, was unique to STS.

**Electronic supplementary material:**

The online version of this article (doi:10.1186/s13229-015-0029-9) contains supplementary material, which is available to authorized users.

## Background

The autism spectrum disorders (ASDs) are heritable neurodevelopmental conditions characterized by impairments in social interaction and communication and restricted, repetitive, and stereotyped patterns of behavior. Several hundred different genetic loci, including single gene mutations, chromosome abnormalities, and copy number variations, have now been associated with a growing number of diagnosed cases [1]. The genetic underpinnings of the remainder of the cases are unclear, but they are postulated as being related to the interactions of multiple genes with environmental factors.

One of the questions central to the study of ASD is how so many different genetic conditions can produce a similar clinical phenotype. Part of the answer to this problem appears to be that a variety of etiologically heterogeneous disorders converge on and disrupt key stages of neurodevelopment that affect specific brain regions and molecular pathways. Evidence for this has come in part from pathway analyses of ASD candidate genes and changes of gene expression in brain tissue [2, 3]. Several genomic and gene expression studies of brain tissue have shown distinct and reproducible changes in transcriptome organization [4], genetic pathways related to neuron function and development [5–7], and immune pathways [5] in ASD.

Given that changes in gene expression must ultimately underlie the clinical phenotype at least to some degree, what might be driving these changes that appear to be region and perhaps even cell specific? Among the many factors that can regulate gene expression—including DNA methylation, transcription factors, and others—we opted to examine small non-coding RNA (sncRNA) in this study, including microRNA (miRNA) and small nucleolar RNA (snoRNA). miRNA are of particular interest because, although they do not code for protein, they bind sequence-specific sites in target transcripts to regulate expression levels of mRNA and/or modulate protein translation [8]. There is growing evidence suggesting miRNA may play a role in ASD supported through mining of SNP and CNV data [9, 10], epidemiological studies of genetic variants [11], and serum biomarker studies [12, 13]. It is estimated that over 60 % of all genes are regulated by miRNA because each miRNA has dozens to hundreds of targets. For this reason, we postulated that miRNA could play an important role in regulating the transcriptional changes that have been reported in ASD brain. Moreover, because miRNA (and other small and non-coding RNA) are often cell or tissue specific and are important in timed molecular events, they can play critical roles in very specific aspects of neurodevelopment [8]. Disturbances in a single miRNA could affect hundreds of genes, directly, and many hundreds of genes, indirectly, and would be one way where a single disturbance could dramatically affect complex cellular organization and function.

Recent whole genome expression studies show that gene expression in brain tissue is region specific and can reflect the distributions of major cell types including neurons and glia [14]. In cases with no known developmental disorders, neocortical transcriptional patterns are relatively homogenous and physically closer regions typically exhibit more similar transcriptomes. However, the primary sensory cortices appear to show the most distinct transcriptional profiles [14], suggesting that they are transcriptionally as well as functionally distinct from other cortical subtypes. To begin to address the underlying genetics of neuropathology, examination of transcriptionally distinct brain regions may be crucial in developing a precise understanding of developmental or pathological disturbances in regulatory mRNA modulators like miRNA.

To examine the expression of the small non-coding RNAs in ASD versus typically developing brains, we focused on two functionally distinct but physically close areas in the temporal lobe, the superior temporal sulcus (STS) and the primary auditory cortex (PAC). The STS is instrumental in functions critical for reciprocal social interaction [15–17] and language [18–20] that are intimately related to core features of ASD [15]. Abnormalities in the STS have been detected in ASD including decreased gray matter, hypo-perfusion at resting state, and abnormal activation during social tasks [21, 22]. In contrast, the PAC is a primary sensory cortex that processes rudimentary components of sounds, e.g., auditory tone, and may function similarly in ASD and control subjects [23]. As such, we predicted that miRNA in PAC would be less affected in ASD than STS. Through careful dissection of these two regions, isolation and examination of expressed sncRNA, identification of regulated gene targets, and functional interpretation of RNA, we found, contrary to our expectation, miRNA in both regions were altered, while no single miRNA was commonly affected in both regions.

## Methods

### Brain tissue

**Fig. 1:**
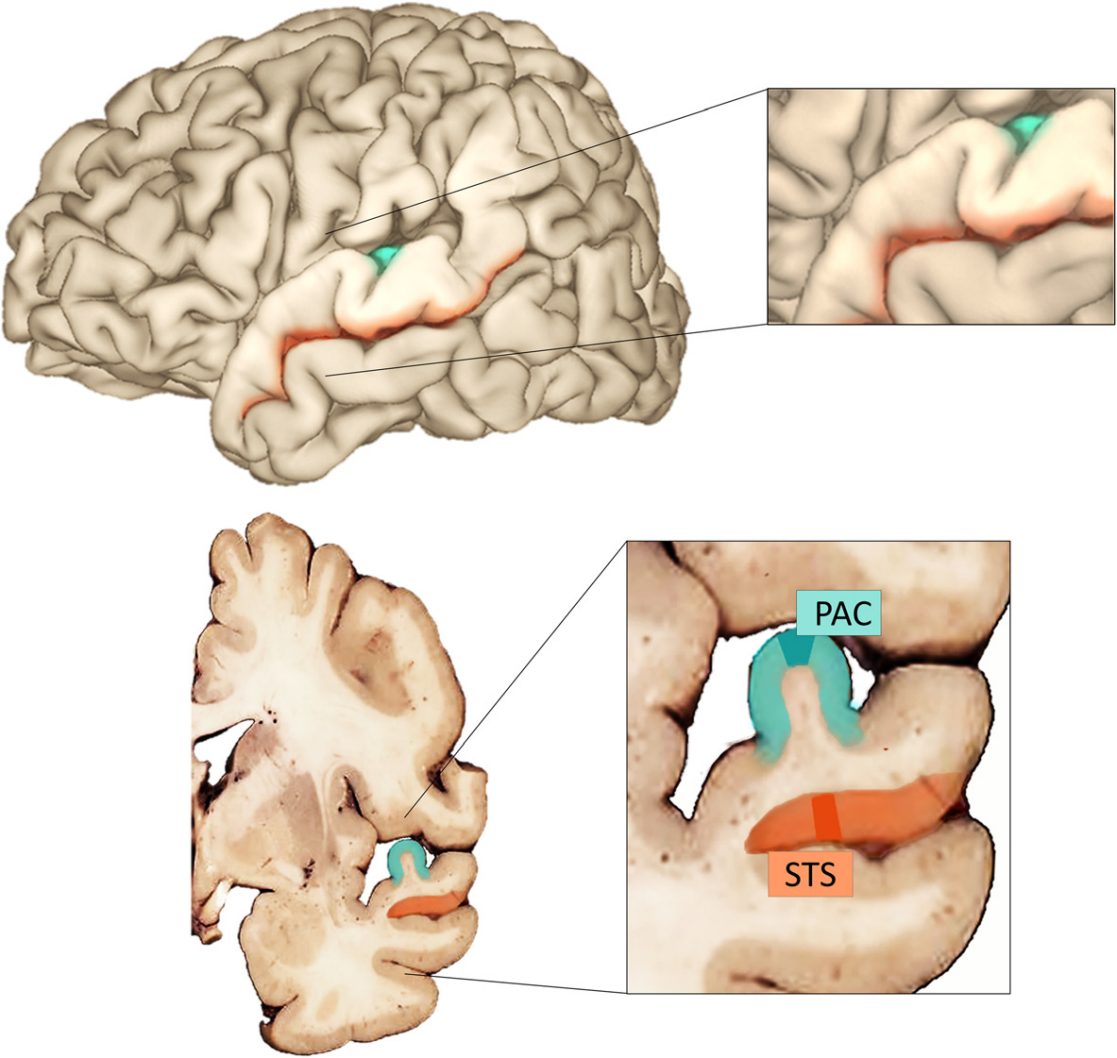
Sampling of brain regions. Schematic of the human brain indicating regions of the superior temporal gyrus sampled—superior temporal sulcus (STS - *orange*) and primary auditory cortex (PAC - *teal*). A section from the caudal portion of the temporal lobe was utilized for each case (the approximate position is noted by the dashed lines). In the same coronal section, tissue blocks for the PAC (*dark teal*) were sampled from Heschl’s gyrus (*light teal*) and blocks for the STS (*dark orange*) were sampled from the upper wall of the STS (*light orange*)

This study was exempt from human subjects review by the Internal Review Board at the UC Davis School of Medicine. Donor brain tissue was obtained from the Autism Tissue Program collection previously housed at the Harvard Brain Tissue Resource Center (http://www.mcleanhospital.org/research-programs/harvard-brain-tissue-resource-center). Postmortem Confirmation of Consent was provided by next-of-kin and held along with identifiable personal health information by the Autism Tissue Program. A total of 36 samples were obtained from ten ASD and eight control subjects. Individual and summarized subject demographics are presented in Table 1. One STS and one PAC sample were taken from the same frozen coronal brain section of each donor brain. The primary auditory cortex (PAC) sample included the primary sensory areas of Brodmann’s areas 41 and 42 and was taken from the crown of Heschl’s gyrus (Fig. 1), where it is reliably found as indicated by functional and cytoarchitectonic maps [24]. Samples from the superior temporal sulcus (STS) included the polymodal association cortex of Brodmann’s area 22 and were taken from the upper wall of the superior temporal sulcus immediately opposite Heschl’s gyrus [25, 26] (Fig. 1).

**Table 1:** Individual demographic information for the brain donors of samples used in this study and postmortem interval of samples

Primary diagnosis	Case number	Sex	Age (years)	Diagnostic measure	PMI (hours)	Primary cause of death
ASD	B-7002	F	5	ADI-R	33.0	Drowning
ASD	B-5342	F	11	ADI-R	12.9	Drowning
ASD	B-7575	M	15	Suspected ASD	30.8	Head trauma
ASD	B-6640	F	29	ADI-R	17.8	Seizure/stroke
ASD	B-7762	M	30	Suspected ASD	22.9	Epilepsy
ASD	B-5173	M	30	ADI-R	20.3	Gastrointestinal bleeding/seizure
ASD	B-6401^a^	M	39	ADI-R	14.0	Cardiac tamponade
ASD	B-7085	F	49	Suspected ASD	21.1	Cancer
ASD	B-7376^b^	F	52	ADI-R	39.2	Unknown
ASD	B-7886	M	50	ADI-R	22.7	Aspiration/seizure
ASD mean	*n* = 10		31.0 ± 5.3		23.5 ± 2.7	
CTRL	B-6736	F	4	-	17.0	Acute bronchopneumonia
CTRL	B-7387	M	17	-	30.8	Asphyxia/hanging
CTRL	B-7738	M	24	-	35.5	Unknown
CTRL	B-7369	M	36	-	26.0	Possible pulmonary embolism/MI
CTRL	B-7835	F	39	-	25.3	Asphyxia/pneumonia
CTRL	B-7333	M	40	-	25.3	Hepatic encephalopathy
CTRL	B-8018	M	54	-	19.9	Unknown
CTRL	B-8155	M	58	-	20.5	Unknown
CTRL mean	*n* = 8		34.0 ± 6.5		25.0 ± 2.1	

Demographics of each brain donor. The average ± the standard error of the mean is provided for ASD and CTRL. There were no significant differences in sex, age, or PMI between ASD and CTRL groups

*ASD* autism spectrum disorders, *CTRL* typically developing control, *PMI* postmortem interval

^a^STG excluded

^b^PAC excluded

### RNA isolation and arrays

Total RNA was isolated from ~10 to 75 mg of dissected brain tissue using the RecoverAll Total Nucleic Acid Isolation Kit (Ambion) with omission of the heat de-paraffinization step. Yields of RNA were an average of 200 ng/mg brain tissue. Quality of RNA was assessed using the Agilent 2100 Bioanalyzer with the RNA 6000 Nano kit, and RNA quantity was measured using the NanoDrop ND-1000. Samples had A260/A280 absorbance ratios ≥1.6 [27, 28], and RNA integrity numbers ranged between 2.2 and 5.3.

Two hundred nanograms of total RNA were processed for Affymetrix miRNA 3.0 microarrays according to the manufacturer’s protocol and our previous publication [28]. These arrays contain probes for 5607 small non-coding RNA including 1733 mature human miRNA, 1658 human pre-miRNA, and 2216 human snoRNA, CDBox RNA, H/ACA Box RNA, and scaRNA. Briefly, 200 ng of RNA is ligated to a biotinylated marker using the FlashTag Biotin HSR RNA labeling kit (Affymetrix). No amplification of the RNA was required during this procedure. The samples are hybridized to the microarrays, and biotin is detected with streptavidin-PE. The microarrays are scanned and fluorescent signals measured and transferred into a CEL file for subsequent analyses. Standard quality control measures are applied, such as excluding arrays that are significant outliers or showing skewing in the overall intensity images. Two samples were excluded after these tests (see Table 1). All within chip quality control hybridization metrics on remaining samples were within normal ranges indicating successful sample processing.

### Statistics

Analyses were performed using Partek Genomics Suite 6.6 (Partek Inc., St. Louis). *Cel* files were imported and normalized using Robust Multiarray Averaging (RMA) [29]. The primary comparisons were expression differences of each sncRNA for ASD versus control cases in the PAC and expression differences of each sncRNA for ASD versus control in the STS. Age and postmortem interval (PMI) were included in the following statistical model: Y_*ijk*_ = *μ* + Primary Diagnosis_*i*_ + age + PMI + Region_*j*_ + Primary Diagnosis*Region_*ij*_ + *ε*_*ijk*_, where Y_*ijk*_ represents the *k*^th^ observation on the *i*^th^ Primary Diagnosis *j*^th^ Region, *μ* is the common effect for the whole experiment, and *ε*_*ijk*_ represents the random error component.

Statistical significance was defined as *P* ≤ 0.005 combined with an absolute fold change ≥1.2. Inclusion of a fold change threshold helps identify changes of biological significance [27, 30, 31].

### Unsupervised hierarchical clustering

Unsupervised hierarchical clustering was performed in the Partek Genomics Suite in order to visualize differences of sncRNA expression between ASD and controls. Hierarchical clustering grouped similar sncRNA expression levels and samples into “clusters” using Euclidean dissimilarity and average linkage method and drawing a tree (dendrogram) that shows the hierarchy of the clusters.

### Pathway analysis

In order to examine the functional significance of the altered miRNA, computationally derived targets of the miRNA were determined. These predicted mRNA targets were then included in an over-representation pathway analysis to determine biological functions from the Kyoto Encyclopedia of Genes and Genomes (KEGG). This was done using the web-based software tool DIANA miRPath v2.0 [32]. Target genes of miRNA lists were computationally predicted using the DIANA microT-CDS with threshold set to 0.9. These mRNA targets were then used to determine if there were more represented genes in a given biological pathway than would be expected by chance using a Fisher exact test corrected for multiple comparisons (FDR, *P* < 0.05) [32].

In order to contrast the biological and functional significance of our study with studies previously reporting gene or gene expression lists associated with autism spectrum disorder, we performed functional over-representation analysis on KEGG pathways using reported data from two prior analyses of temporal cortex in ASD, Voineagu et al. (135 probes) [4] and Garbett et al. (152 probe sets) [33], and two datasets broadly focusing on ASD-associated genes, Pinto et al. (139 genes) [34] and the SFARI database of positively associated ASD genes (573 genes) [35]. Datasets were input into the online WebGestalt tool [36] to derive the KEGG pathway data for Fig. 5. Predicted gene targets from our study were taken from microT-CDS after combining all brain samples (STS and PAC) and then deriving miRNA that were different between ASD and CTRL groups (*P* < 0.005, fold change > |1.2|). Combining temporal regions allowed for a more appropriate comparison to the previous studies that did not target specific functional territories. Euler diagrams of area proportional overlap were generated using eulerAPE [37].

## Results

### Small non-coding RNA significantly differed between ASD and controls

**Fig. 2:**
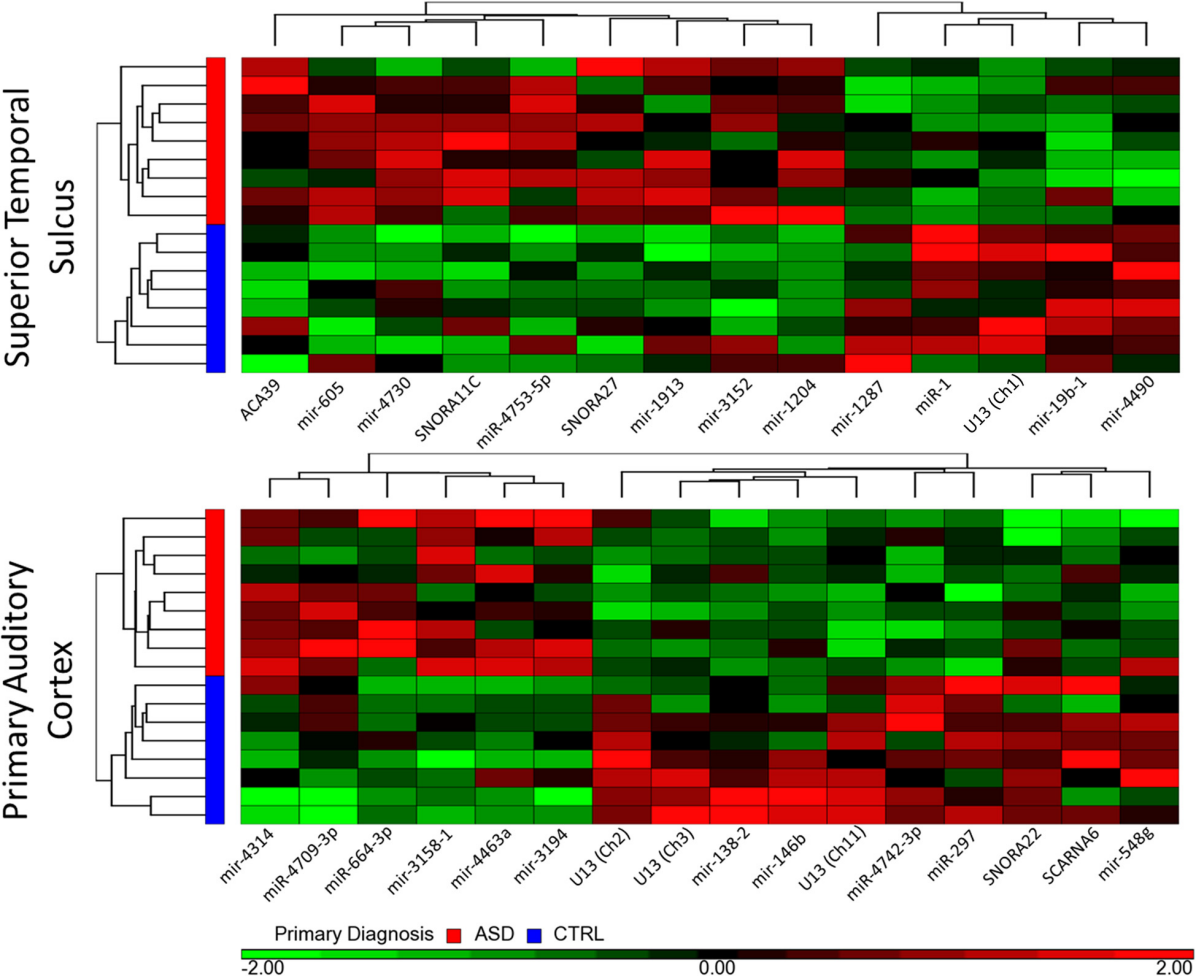
Relative expression of sncRNA in STS and PAC of ASD and CTRL subjects. Heat maps showing hierarchical clustering of dysregulated small non-coding RNAs on the *X axis* for ASD and control (CTRL) subjects on the *Y axis* for the superior temporal sulcus (STS) (*upper panel*) and the primary auditory cortex (PAC) (*lower panel*). Note that the ASD and CTRL subjects cluster together in both the PAC and STS. *Bright red color* indicates a relative twofold increase in expression for the particular sncRNA, and a *bright green color* indicates a relative twofold decrease in expression across ASD and control subjects (see *bar at bottom of lower panel*)

There were no significant differences in the sex or age of ASD compared to control subjects (*P* > 0.05, Table 1). Table 2 lists the sncRNA that were differentially expressed in ASD compared to controls (*P* < 0.005 and fold change > |1.2|) in the temporal lobe for the superior temporal sulcus (STS) and for the primary auditory cortex (PAC) (detailed fold change and *P* values provided in Additional file 1: Tables S1 and S2). When subjected to hierarchical clustering, these sncRNA completely separated ASD from control subjects for both the PAC and STS brain regions (Fig. 2), lending further support that these sncRNA were significantly different in ASD compared to controls in both brain regions.

**Table 2:** Differentially expressed sncRNA between ASD and CTRL

Class of sncRNA	Superior temporal sulcus	Primary auditory cortex
miRNA	↑ miR-4753-5p	↑ miR-664-3p, miR-4709-3p
	↓ miR-1	↓ miR-297, miR-4742-3p
snoRNA	↑ ACA39, SNORA11C, SNORA27	↑ (none)
	↓ U13 (Chr1)	↓ SNORA22, U13 (Chr2), U13 (Chr3), U13 (Chr11), SCARNA6
Stem-loop precursor miRNA	↑ mir-1204, mir-1913, mir-605, mir-3152, mir-4730	↑ mir-3158-1, mir-3194, mir-4314, mir-4436a
	↓ mir-1287, mir-19b-1, mir-4490	↓ mir-138-2, mir-146b, mir-548 g

Small non-coding RNA (sncRNA) that had significant changes in expression in the superior temporal sulcus (STS) and primary auditory cortex (PAC) of ASD brains compared to control brains (*P* < 0.005 and fold change > |1.2|)

*miRNA* microRNA, *sncRNA* small non-coding RNA, *snoRNA* small nucleolar RNA

**Fig. 3:**
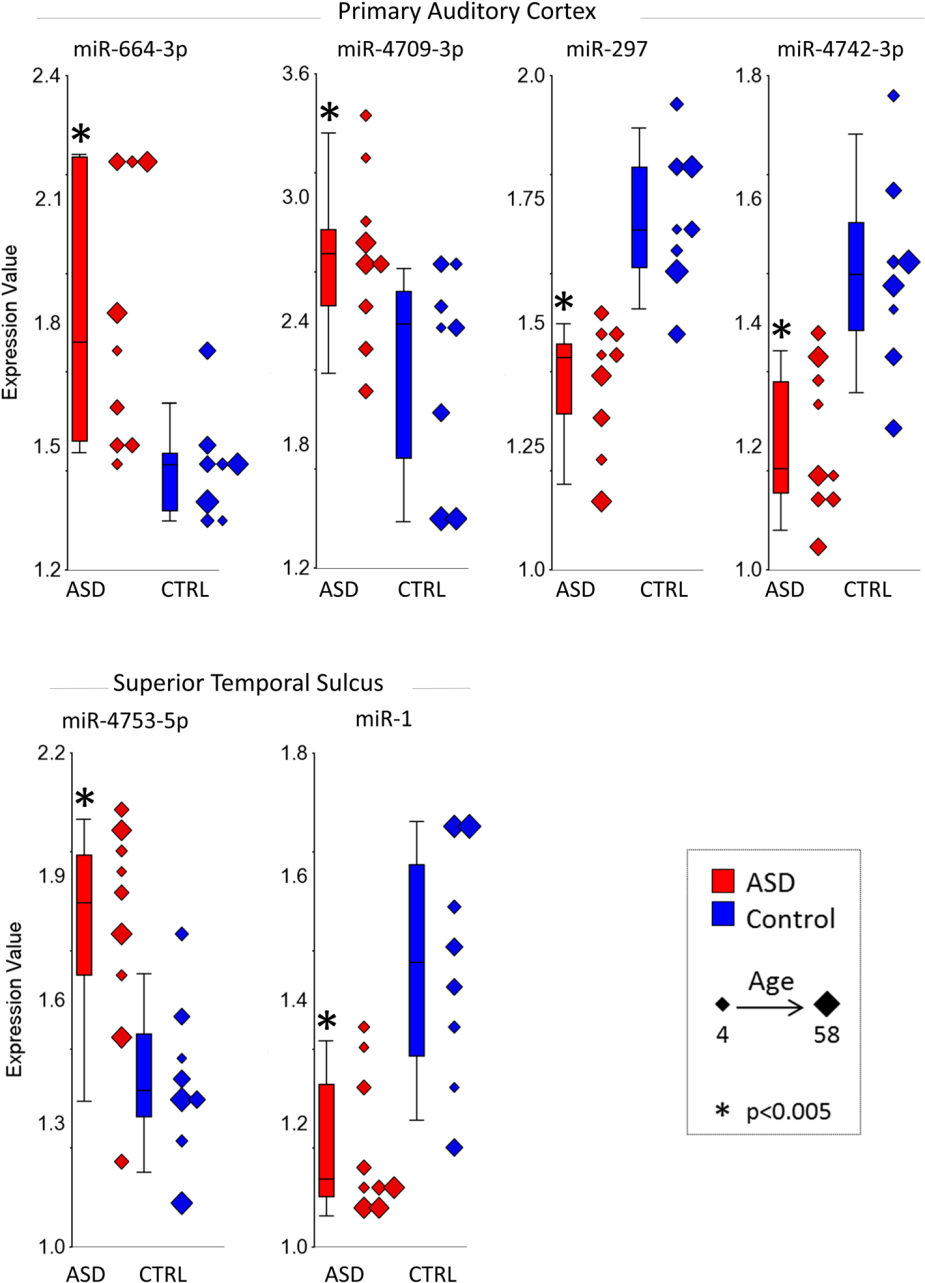
Expression levels of significant miRNA is STS and PAC. Expression levels of microRNA (miRNA) that had significantly increased or decreased expression in ASD compared to control subjects in the superior temporal sulcus and in the primary auditory cortex (*P* < 0.005 and fold change > |1.2|). ASD values are in *red*, and control (CTRL) values are in *blue*. Note that individual subjects are plotted along with *box* and *whisker plots*, and the size of the *diamonds* for each individual corresponds to age of the subject

For the STS, there were 14 sncRNA differentially expressed between ASD and control brains (Table 2). Of the two mature miRNA, miR-4753-5p increased expression and miR-1 decreased expression (Fig. 3, Table 2). For the PAC, there were 16 sncRNA differentially expressed between ASD and control brains (Table 2). Of the four mature miRNA, miR-664-3p and miR-4709-3p increased expression and miR-297 and miR-4742-3p decreased expression (Fig. 3, Table 2).

Small nucleolar RNA (snoRNA), another class of small non-coding RNA, were also differentially expressed between ASD and control brains. ACA39, SNORA11C, and SNORA27 increased in STS while the U13 paralog on chromosome 1 decreased expression in STS (Table 2). In PAC, no snoRNA had increased expression, whereas SNORA22, SCARNA6, and three U13 paralogs on chromosomes 2, 3, and 11 showed decreased expression in ASD compared to controls (Table 2). Eight pre-miRNA changed expression in the STS, and seven pre-miRNA changed expression in the PAC in ASD compared to controls (Table 2).

### Functional roles of miRNA in the STS and PAC

**Fig. 4:**
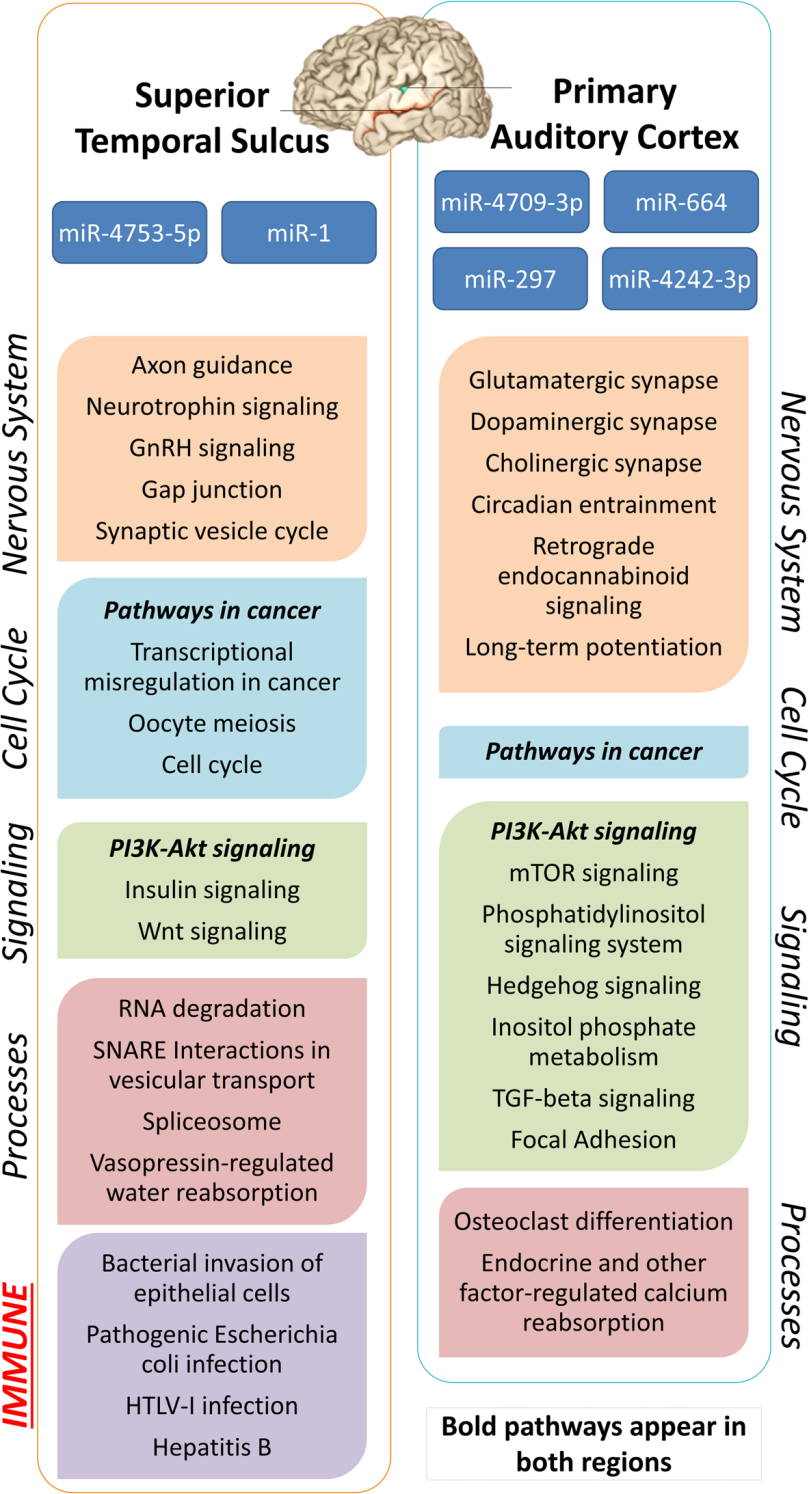
Functional categorization of miRNA target genes. KEGG pathways of the predicted gene targets of the microRNA that showed altered expression in ASD compared to control subjects in the PAC and in the STS. These pathways had more predicted regulated target genes in the pathway than would have been expected by chance (*P* < 0.05). Pathways are grouped according to categories in vertical text. Only miRNA differentially expressed in STS had target genes over-represented in immune related pathways and functions

Based on the microT-CDS algorithm (which includes coding and 3′ UTR sequences, threshold = 0.9), we found miRNA differentially expressed in ASD had 1214 predicted gene targets in PAC and 445 in STS (Additional file 1: Table S3). Over represented KEGG pathways that contained more gene targets than expected by chance (Fisher exact test, FDR corrected *P* < 0.05) are summarized in Fig. 4. KEGG pathways regulated by these miRNA showed little overlap between regions (Fig. 4). Indeed, only two pathways were shared between PAC and STS: “pathways in cancer”, which are cell cycle, cell differentiation, mitochondrion, and gene regulation focused, and the PI3K-Akt signaling pathway (Fig. 4). Another major finding is that immune pathways under control of the differentially expressed miRNA appear to be dysregulated only in STS whereas neuronal, cell cycle, signaling and cell processes were dysregulated in both PAC and STS in ASD compared to control subjects (Fig. 4).

**Fig. 5:**
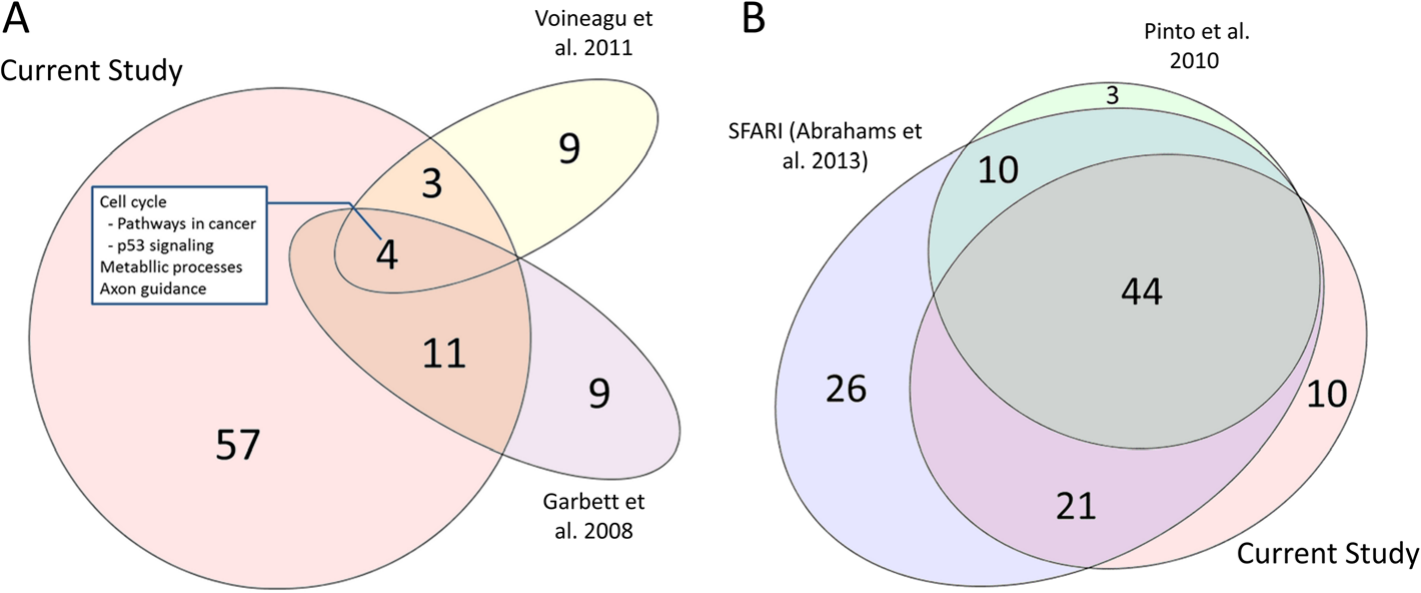
Functional similarities to other studies looking at ASD-related genes. Overlap of KEGG pathways identified through analysis of mRNA targets of brain miRNA in ASD and control brain from the STS and PAC combined analysis with previously published and compiled gene lists associated with ASD in (**a**) studies with transcriptional measurements of ASD brain (Garbett et al. [33] and Voineagu et al. [4]) and (**b**) sets of genes associated with ASD (Pinto et al. [34] and Abrahams et al. (SFARI) [35]). Numbers indicate KEGG pathways significantly over-represented by mRNA in the respective data set. The 44 KEGG pathways overlapping between this study, SFARI, and Pinto et al. appear in Additional file 1: Table S4

Figure 5 shows that many of the predicted brain miRNA-regulated pathways in the STS and PAC-combined analysis in this study were also predicted to be dysregulated based on genes identified in the expression studies of Voineagu et al. [4] and Garbett et al. [33] as well as pathways implicated in ASD gene sets from Pinto et al. [34] and the SFARI gene database [35]. The significance of the overlap of our data to each of these data sets was *P* < 0.05 using the hypergeometric probability function *phyper* in R.

## Discussion

This study provides the first evidence of alterations in expression of specific small regulatory non-coding RNA in the temporal cortex of autism spectrum disorder (ASD) brains. The major findings include the following: 1) There are differences in miRNA, snoRNA, and precursor miRNA in both the association cortex of the superior temporal sulcus (STS) and the primary auditory cortex (PAC). While the STS is involved in functions associated with the core features of ASD, the many changes in sncRNAs in primary auditory cortex were not expected as it is not functionally associated with core deficits of ASD. 2) Functional pathways for the gene targets of dysregulated miRNA across both regions were associated with nervous system, cell cycle, cell signaling, and other processes. 3) miRNAs that regulate immune functions were only altered in STS, supporting a role for immune dysfunction in STS associated with the core social deficits of autism. Even a few dysregulated miRNA and other sncRNAs have the potential to affect transcription and/or translation of hundreds of target genes and affect brain signaling, connectivity, and behavior.

### Differentially expressed sncRNA in both primary and association cortex in ASD

As hypothesized, based on functional and structural differences known to be present in ASD STS, we found that the STS contained differentially expressed regulatory sncRNA. One mature miRNA, miR-4753-5p, exhibited increased expression levels and another, miR-1, had decreased expression in ASD relative to control tissue. Five immature, stem-loop precursor miRNA showed increased expression, and three had decreased expression in the STS. Given their regulatory role, miRNA can impact hundreds of gene targets and can greatly influence expression levels of mRNA and protein translation [8], potentially making a substantial contribution to differences in overall gene expression levels in ASD.

There were also differences in a number of small nucleolar RNAs (snoRNAs) in STS in ASD. snoRNAs are small RNA molecules that act to guide chemical modifications of other RNAs including ribosomal RNAs, transfer RNAs, and small nuclear RNAs. Three snoRNAs, including ACA39, SNORA11C, SNORA27, exhibited increased expression, and one, U13 (Chr1), had decreased levels. Functionally, sequences within snoRNAs target them to specific mRNA targets, with the C/D box snoRNAs methylating target bases and H/ACA box snoRNAs being associated with pseudouridylation of target bases. These modifications likely affect translation as well as RNA silencing, telomerase maintenance, and alternative splicing [38]. For example, U13 is involved in proper formation of the 3′ end of the 18S ribosomal subunit [39], potentially impacting protein translation and alternative splicing. Aberrant expression of U13 could have widespread cellular effects in the brain. We find that non-coding snoRNAs along with non-coding miRNA are dysregulated in ASD brain, while other studies have also implicated long non-coding RNAs in ASD neuropathology [40, 41].

To our knowledge, there have not been any previous studies targeting molecular abnormalities specifically in primary sensory cortex of ASD brains, making this the first report suggesting regulatory abnormalities in a primary sensory region. In PAC, two mature miRNAs exhibit increased expression, miR-664-3p and miR-4709-3p, and two decreased expression, miR-4742-3p and miR-297. Five snoRNAs showed decreased expression, including SNORA22, SCARNA6, and three paralogs of U13. Four stem-loop precursors were additionally up-regulated, while three had decreased expression.

It is notable that three of the identified precursor miRNA have been previously associated with ASD. miR-4436 has been described as a candidate susceptibility miRNA gene in autism [42]. miR-548 is dysregulated in ASD lymphoblastoid cell lines [43] and targets genes associated with several pathways implicated in ASD including ubiquitin, Wnt, axon guidance, LTP, and natural killer cells [30]. Additionally, it can regulate host antiviral responses via direct targeting of IFN-λ1 [44], which may have relevance for maternal immune activation models of ASD and other neurodevelopmental disorders [45, 46]. miR-146 is reported to have altered expression in ASD cerebellar cortex [47] and in ASD lymphoblastoid cell lines. In the nervous system, miR-146 (a,b) modulates AMPA receptor endocytosis [48] and glial proliferation via Notch signaling [49]. miR-146 also influences the activation state of brain microglia [50]. Microglia are reported to increase in number, show evidence of activation, and show increased gene expression in ASD post-mortem brain tissue [51–55]. Moreover, deficiencies in neuron-microglia signaling result in impaired functional brain connectivity and can influence social behavior [56]. Although the functional importance of immature miRNA is not clear, the mature forms of the three stem-loop precursors found in the PAC have previously been associated with ASD.

A number of the predicted gene targets of the miRNA we found different between ASD and typically developing STS have previously been identified in studies looking at gene expression in the temporal cortex [4, 33]. These target genes (INHBA, WDR1, TAGLN2, FNDC3B, ZFP36L1, CNN3, CPLX2, ZNF365, and GLS2) have important roles including those related to “cell communication and motility” and “cell fate and differentiation.” As we observed decreased miR-1 expression and increased miR-4753-5p expression in our study, we would expect to find higher and lower expression of the respective gene transcripts they target in STS. The regulation of these genes and others by altered patterns of miRNA may be at least in part responsible for regional and cellular differences that shape the social and behavioral phenotypes associated with ASD.

Dysregulation of sncRNAs in the PAC of ASD brain is a novel molecular finding we did not predict. Although sensory dysfunction is not considered a core diagnostic feature, neurophysiological studies have reported auditory abnormalities in ASD patients. Abnormalities in cortical auditory-evoked potentials have been reported when sound is presented to the left ear [57]. In contrast, speech stimulation does not produce fMRI abnormalities in PAC of individuals with ASD [23]. Atypical vertical sound localization and sound-onset sensitivity evidenced in ASD cases likely involves primary auditory areas [58]. In addition, a magnetoencephalography study examining differences in auditory cortex function in young ASD and TD children found that there is atypical brain function in the auditory cortex in young children with ASD, regardless of language development [59]. Coupled with neurophysiological findings, the dysregulated sncRNA in PAC in this study provide additional evidence for PAC dysfunction in ASD. Increased attention to primary cortices may be warranted in future investigations of ASD, as such dysfunction could conceivably contribute to cognitive impairments in complex social and linguistic tasks.

### miRNA targets incorporated into pathways associated with ASD pathogenesis

We postulated that miRNA play an important role in regulating the transcriptional changes that have been reported in ASD brain tissue. Several genomic and gene expression studies of ASD brain have shown distinct and reproducible differences from typically developing cases that involve neurons/synapses and the immune system. In one study, Voineagu et al. showed consistent differences in transcriptome organization between ASD and typically developing brain by gene co-expression analysis [4]. Moreover, two main modules of co-expressed genes in ASD brain were identified including a neuronal/synapse module that was associated with genes identified by GWAS in ASD and an immune module that was not associated with ASD GWAS genes [4, 60]. These data supported prior studies of neuron, glia, and immune abnormalities in ASD brain [33, 51, 61–67]. Another recent whole genome study showed age-related differences of gene expression in ASD brain that also affected neuron and immune pathways including neurogenesis, cell cycle, cell differentiation, cell death, and immune response in young ASD brain [5]. A recent study used weighted gene co-expression network analysis (WGCNA) combined with ASD candidate genes to suggest that cortical projection neurons in layers V and VI of prefrontal/frontal cortex during 10–24 weeks post-conception were key cells in ASD pathogenesis [6]. In a similar WGCNA analysis using different ASD candidate genes, the superficial (pyramidal) glutamatergic cortical projection neurons were implicated in ASD pathogenesis [7].

We found many pathways associated with differentially expressed miRNA in ASD temporal cortex were similarly associated with nervous system and immune function, as well as basic cellular processes previously reported to be dysregulated in ASD. Of the 34 predicted pathways and processes that were represented in the target genes of miRNA in STS and PAC (Fig. 4), a number have been implicated in previous ASD studies including glutamatergic, dopaminergic, and cholinergic synapse pathways, axon guidance, synaptic vesicle cycling, neurotrophins, immune pathways, hedgehog and Wnt signaling, spliceosome, and RNA degradation [2–4, 27, 60]. Comparing our broader temporal cortex findings with previous analyses, it is notable that 44 of the 75 pathways regulated by differentially expressed miRNA in our study are shared with the 57 pathways reported to be affected by copy number variants in ASD [34] (Fig. 5, Additional file 1: Table S4). Moreover, we found 7 and 15 pathways, respectively, are shared with the 16 and 24 pathways reported to demonstrate alterations in the two prior analyses of mRNA expression ASD temporal cortex [4, 33] (Fig. 5). Common functions associated with pathways across all studies of ASD temporal cortex include cell cycle, metabolic, and neuron-related pathways and processes. Given the substantial number of convergent pathways between studies of temporal cortex mRNA and miRNA expression, it is highly plausible that miRNA are exerting a regulatory influence over mRNA associated with atypical cortical function in ASD.

Within the temporal lobe, two pathways were shared between PAC and STS, the “PI3K-Akt pathway” and “pathways in cancer.” The PI3K-Akt pathway is of interest as it has been implicated in a number of previous ASD studies. The PI3K-Akt/mTOR pathway is dysregulated in monogenic disorders associated with ASD including Fragile X, tuberous sclerosis, neurofibromatosis, and PTEN [68]. Rare and even some common genetic variants associated with ASD affect PI3K-Akt signaling [2, 69]. Studies of gene expression in blood of ASD subjects have found abnormal PI3K-Akt/mTOR signaling [70]. Our studies of alternative splicing of mRNA expressed in blood leukocytes detected abnormalities of PI3K-Akt signaling in about half the 2–4 year old ASD children with idiopathic ASD [27]. Thus, the current study suggests abnormal PI3K-Akt signaling in temporal cortex of ASD subjects in both primary auditory and STS association cortex, and previous studies suggest PI3K-Akt abnormalities in blood and differential alternative splicing of PI3K-Akt pathways in blood of many ASD subjects. These results would support this as a potential therapeutic target in at least a subgroup of ASD subjects as has been suggested by others [71].

One of the major findings of this study is that few predicted miRNA-regulated pathways were shared between the PAC and STS, despite the fact that they are adjacent cortical structures. Because many pathways are specific for PAC or STS, there is evidence that differential regulation of miRNA, their mRNA targets, and associated pathways can be region specific in ASD.

Differentially expressed miRNA having a significant number of gene targets in immune and most of the cell cycle KEGG pathways (except “pathways in cancer”) were restricted only to STS. Cell cycle genes might be related to neuronal cell death and/or to glial proliferation and perhaps linked to increased microglial and neuron numbers in ASD brain [51–53, 63, 72]. Observed immune pathways in this study, related to both viruses and bacteria, are consistent with many reports of immune pathway activation in ASD brain and blood [4, 5, 33, 60, 73–75]. Since the immune regulated genes in ASD brain were not among the GWAS-associated ASD genes [4], these immune genes might reflect the environmental factor in ASD pathogenesis. ASD is considered to be a complex genetic disorder in which several genes interact with an environmental precipitant. Activation of immune and cell cycle pathways in STS might suggest that this is a region of primary pathology, and lack of immune and cell cycle activation in PAC might suggest gene changes in PAC are secondary to those in STS association cortex because of interconnections between the two regions. At the very least, these findings suggest molecular and immune disruption pathologies present in the temporal association cortex that are not shared with primary sensory cortex. This further emphasizes the importance of assessing molecular abnormalities in distinct cortical regions.

### Limitations of the study

Future studies will need to confirm the miRNA changes discovered here with microarrays using qRT-PCR, though in other studies we have shown most miRNA regulated on arrays can be confirmed using qRT-PCR [28]. In addition, future studies will need to confirm the changes in a separate cohort, to examine mRNA targets of the miRNA and their associated pathways, and to examine other cortical regions which may also be associated with core ASD symptoms. Future studies of primarily young brains are needed to assess changes around the time of diagnosis, though postmortem studies like this by their nature tend to have older subjects. Though age, gender, and other factors were included in the statistical model, some covariates like medications, cause of death, and others could not be included because of the limited number of samples.

## Conclusions

This study provides the first demonstration of dysregulation of sncRNA in superior temporal cortex of ASD brains that could regulate hundreds of target transcripts and many predicted pathways and processes that have been implicated in previous ASD studies. In addition to changes in sncRNA expression in the STS, transcriptional dysregulation in the PAC could play a role in social and language dysfunction in ASD. This has implications for understanding the pathogenesis of ASD and ascertaining whether the “primary ASD pathology” is only in association cortex and whether abnormalities of both primary auditory cortex and STS temporal association cortex contribute to social and language behavioral dysfunction observed in ASD.

## Additional file

Additional file 1: Table S1-S4. Table 1: *P* values and fold changes for the 14 sncRNA different between ASD and CTRL STS. Table S2: *P* values and fold changes for the 16 sncRNA different between ASD and CTRL PAC. Table S3: The 445 predicted gene targets of STS and 1214 predicted gene targets of miRNA in PAC based on the microT-CDS algorithm. Table S4: Pathways common to our study, SFARI ASD implicated genes and Pinto et al. 2010 (the 44 common KEGG pathways of Fig. 5B).

## Abbreviations

ASD: autism spectrum disorders
CNV: copy number variant
GWAS: genome-wide association study
KEGG: Kyoto Encyclopedia of Genes and Genomes
miRNA: microRNA
PAC: primary auditory cortex of temporal lobe
PMI: postmortem interval
sncRNA: small non-coding RNA
snoRNA: small nucleolar RNA
SNP: single nucleotide polymorphism
STS: superior temporal sulcus of temporal lobe
WGCNA: weighted gene co-expression network analysis
